# Muscle Characteristics Obtained Using Computed Tomography as Prognosticators in Patients with Castration-Resistant Prostate Cancer

**DOI:** 10.3390/cancers12071864

**Published:** 2020-07-10

**Authors:** Jongsoo Lee, Jee Soo Park, Ji Eun Heo, Hyun Kyu Ahn, Won Sik Jang, Won Sik Ham, Koon Ho Rha, Young Deuk Choi

**Affiliations:** 1 Department of Urology, Urological Science Institute, Yonsei University College of Medicine, 50-1, Yonsei-ro, Seodaemun-gu, Seoul 03722, Korea; js1129@yuhs.ac (J.L.); sampark@yuhs.ac (J.S.P.); heoji87@yuhs.ac (J.E.H.); sindakjang@yuhs.ac (W.S.J.); uroham@yuhs.ac (W.S.H.); khrha@yuhs.ac (K.H.R.); 2 Department of Urology, Gangnam Severance Hospital, Yonsei University College of Medicine, 211, Eonju-ro, Gangnam-gu, Seoul 06273, Korea; wharang11co@yuhs.ac

**Keywords:** castration-resistant prostate cancer, body composition, computed tomography, survival, prognosticator

## Abstract

Limited studies have investigated the correlation between body composition and prostate cancer outcomes. We analyzed the effect of muscle mass and quality on castration-resistant prostate cancer (CRPC) outcomes. Skeletal muscle index (SMI) and skeletal muscle attenuation (SMA) were measured for 411 patients at the L3 vertebral level using computed tomography at CRPC diagnosis and were dived to low and high groups at the value of median. Analysis of the skeletal phenotypes and age (<70 and >70 years) was performed to evaluate the effect of SMI and SMA. The median survival rates for patients with low and high SMI were 19 and 24 months (*p* = 0.015), and those with low and high SMAs were 15 and 26 months (*p* < 0.001), respectively. In the subgroup analysis by age, SMA was a significant prognosticator in both groups, while SMI was a significant prognosticator only in patients aged >70 years. Patients with low SMA + low SMI had the worst prognosis. Muscle characteristics seems to be a prognosticator in survival of CRPC patients and may be considered in treatment planning.

## 1. Introduction

Recently, the incidence and prevalence of prostate cancer has increased because of various reasons [1]. However, owing to the development of multimodal treatment and advancements in techniques, survival rates have increased [2]. Although some drugs are prescribed at a fixed dose for patients receiving chemotherapy, the dose is often determined based on the body surface area (BSA). However, due to cachexia, sarcopenia, and obesity, varying toxicities and effectiveness are observed among patients with similar BSA ranges. Therefore, various researchers have concluded that the BSA should not be the main factor for dose determination. Many studies were conducted to analyze the association between individual body composition (including muscle and fat mass) and survival rates using various measurement techniques. Notably, the relationship between survival rate and muscle characteristics, sarcopenia and myosteatosis, is a prominent issue for patients with cancer [3]. The degree of sarcopenia and myosteatosis can also be used for better understanding of a patient’s general health status and individualized management. Sarcopenia is defined based on the muscle mass of the entire body, which can be represented by several methods such as dual-energy X-ray absorptiometry, bioelectrical impedance analysis, and muscle indexes calculated from computed tomography (CT) scan. Among those, muscle area measurement at the L3 level by computed tomography is the most widely used method to calculate skeletal muscle index (SMI) or psoas muscle index (PMI). Using this information, studies have attempted to identify patients with cancer cachexia who cannot benefit from multimodal treatment and are more suited for palliative therapy [3]. Myosteatosis, an indicator of muscle quality, can be defined based on the Hounsfield unit (HU) of muscles, which can be represented as skeletal muscle attenuation (SMA). SMA reflects fat disposition in the muscle tissue which becomes a more detailed expression of the muscle. Even with similar muscle mass among patients, low SMA represents lower muscle quality and poor prognosis for elderly patients [4,5,6]. Because of the development of computer technology, research on automation of body composition measurements using computed tomography (CT) is ongoing, with highly accurate results [7,8].

Studies investigating the correlation between body composition and prostate cancer are in progress but not yielding clear conclusions or consensus. Although studies have attempted to understand the impact of SMI or PMI on prognosis in patients with castration-naïve and castration-resistant prostate cancer (CRPC), these are few in number and have reported conflicting results. Furthermore, there are no reports on the effects of myosteatosis, which is represented as SMA in our study, on CRPC outcomes. Patients with CRPC are subjected to multimodal medical treatment and prolonged exposure to androgen deprivation therapy (ADT), which leads to changes in body composition as decrease of SMI and SMA [9,10]. Considering the characteristics of patients with CRPC, their body composition and prognosis must be investigated. Therefore, in this study, we aimed to evaluate the relationship between muscle mass, quality, and survival of these patients. Our study is the first large scaled study to reveal the significance of both sarcopenia and myosteatosis for survival of patients with CRPC

## 2. Results

### 2.1. Patient Characteristics

In total, 411 patients were included in the final analysis. For subgroup analysis, patients were stratified into two age groups, according to the median age of 70 years at CRPC diagnosis, and into four groups based on their skeletal muscle phenotype.

The median patient ages at prostate cancer and CRPC diagnosis were 67 (62–72) years and 70 (65–76) years, respectively. The median values of the body mass index (BMI) and BSA were 24.4 (22.5–26.3) kg/m^2^ and 1.73 (1.64–1.84) m^2^, respectively. According to the BMI cut-off points proposed by the World Health Organization for the Asian populations, 52% of the patients were overweight and 9% were obese in our cohort. At the time of CRPC diagnosis, the patients had metastatic lesions in the bones, lymph nodes, and visceral organs, which occurred in 84%, 47%, and 17% of patients, respectively. The median prostate-specific antigen (PSA) level at CRPC diagnosis was 44.2 (12.8–158.0) ng/mL. The 1-, 2- and 3-year survival rates from the time since CRPC diagnosis were 68%, 37%, and 24%, respectively.

### 2.2. Muscle Measurements

The median SMI and SMA values in our cohort were 45.2 (40.6–50.0) cm^2^/m^2^ and 32.4 (28.5–36.5) HU, respectively. When comparing the clinicopathological characteristics, both the high and low SMI and SMA groups showed significant differences in terms of age; levels of hemoglobin (Hb), alkaline phosphatase (ALP), and albumin; visceral metastasis at CRPC diagnosis, 1- and 2-year overall survival rates since CRPC diagnosis; and treated docetaxel cycles. The intermuscular fat area (cm^2^), Charlson comorbidity index (CCI), PSA level at CRPC diagnosis, period of ADT administration before CRPC diagnosis, clinical trial enrollment, and three-year survival since CRPC diagnosis showed significant differences only between the low and high SMA groups. In contrast, only BMI > 23 kg/m^2^ and BSA showed significant differences between the high and low SMI groups (Table 1).

**Figure 1:**
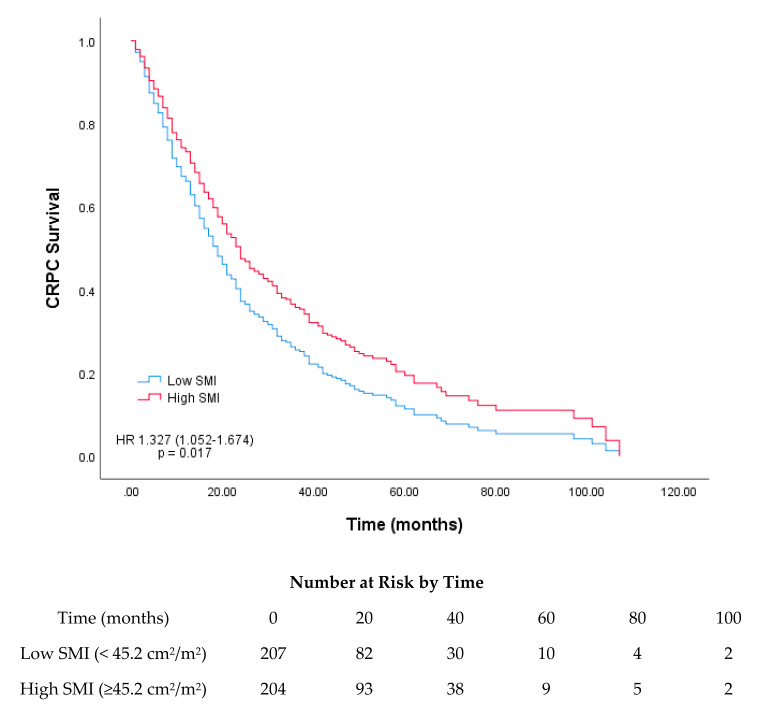
Survival curves based on Cox regression analysis of the survival of patients with castration-resistant prostate cancer (CRPC) stratified according to their median skeletal muscle index (SMI; cm^2^/m^2^).

**Figure 2:**
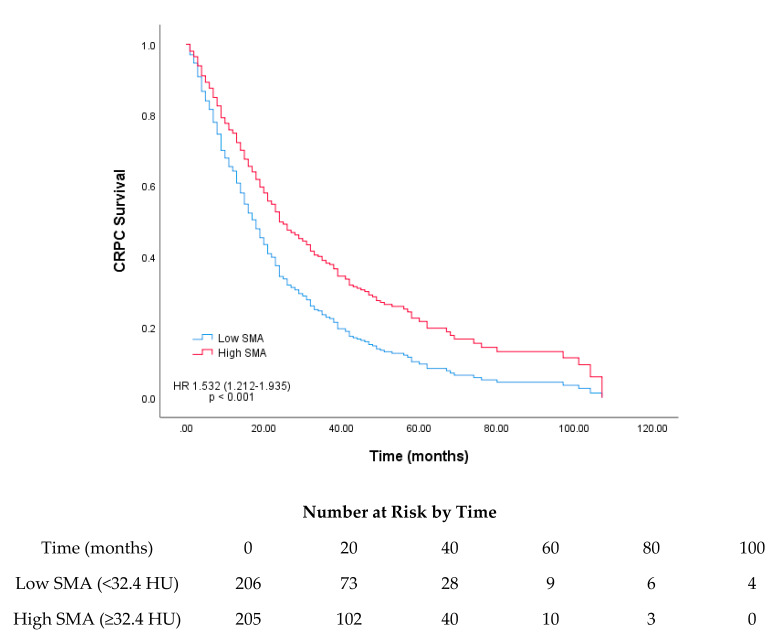
Survival curves based on Cox regression analysis of the survival of patients with castration-resistant prostate cancer (CRPC) stratified according to their median skeletal muscle attenuation (SMA; Hounsfield unit).

Based on the median SMI and SMA, survival curves were plotted using Cox regression analysis for the low and high SMI and SMA groups. The median survival rates for the low and high SMI groups were 19 and 24 months, respectively, and those for the low and high SMA groups were 15 and 26 months, respectively (*p* = 0.017 and *p* < 0.001, respectively; Figure 1 and Figure 2).

**Figure 3:**
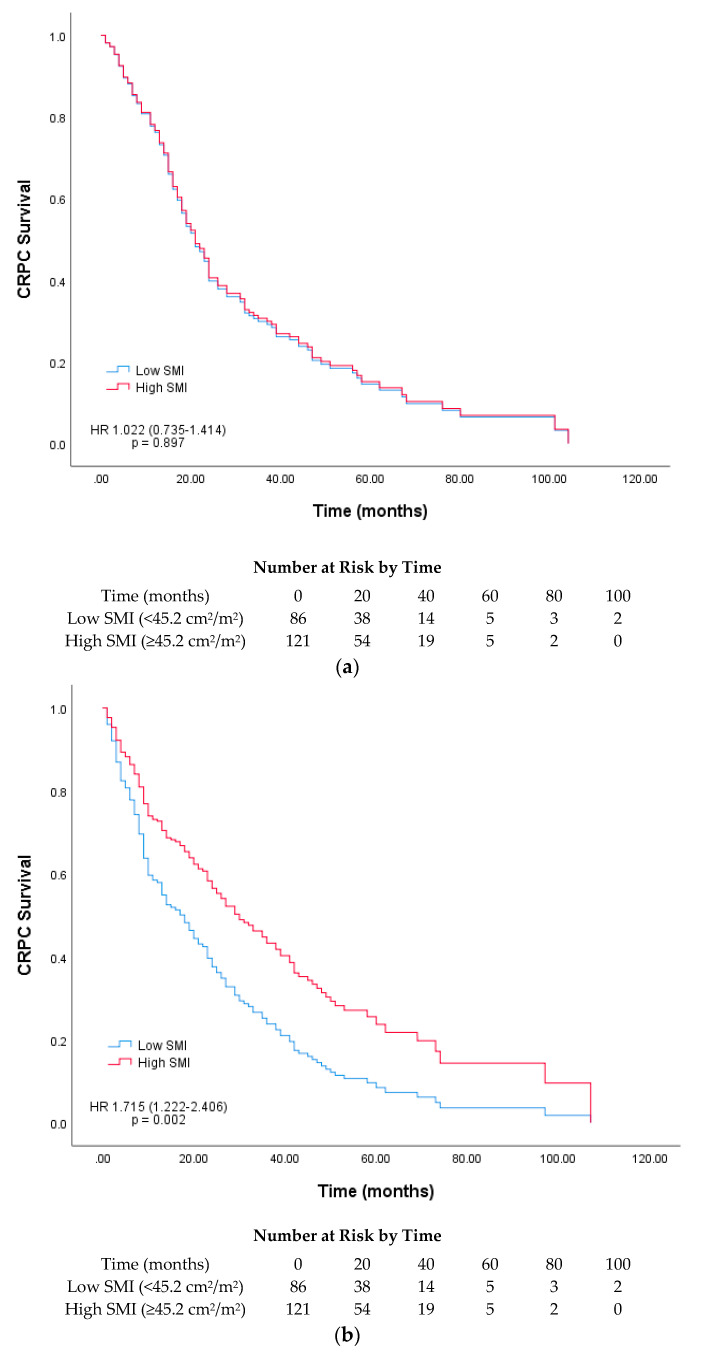
Survival plot based on Cox regression analysis of castration-resistant prostate cancer (CRPC) survival according to the median skeletal muscle index (SMI; cm^2^/m^2^) for the age groups (**a**) <70 and (**b**) >70 years at CRPC diagnosis.

**Figure 4:**
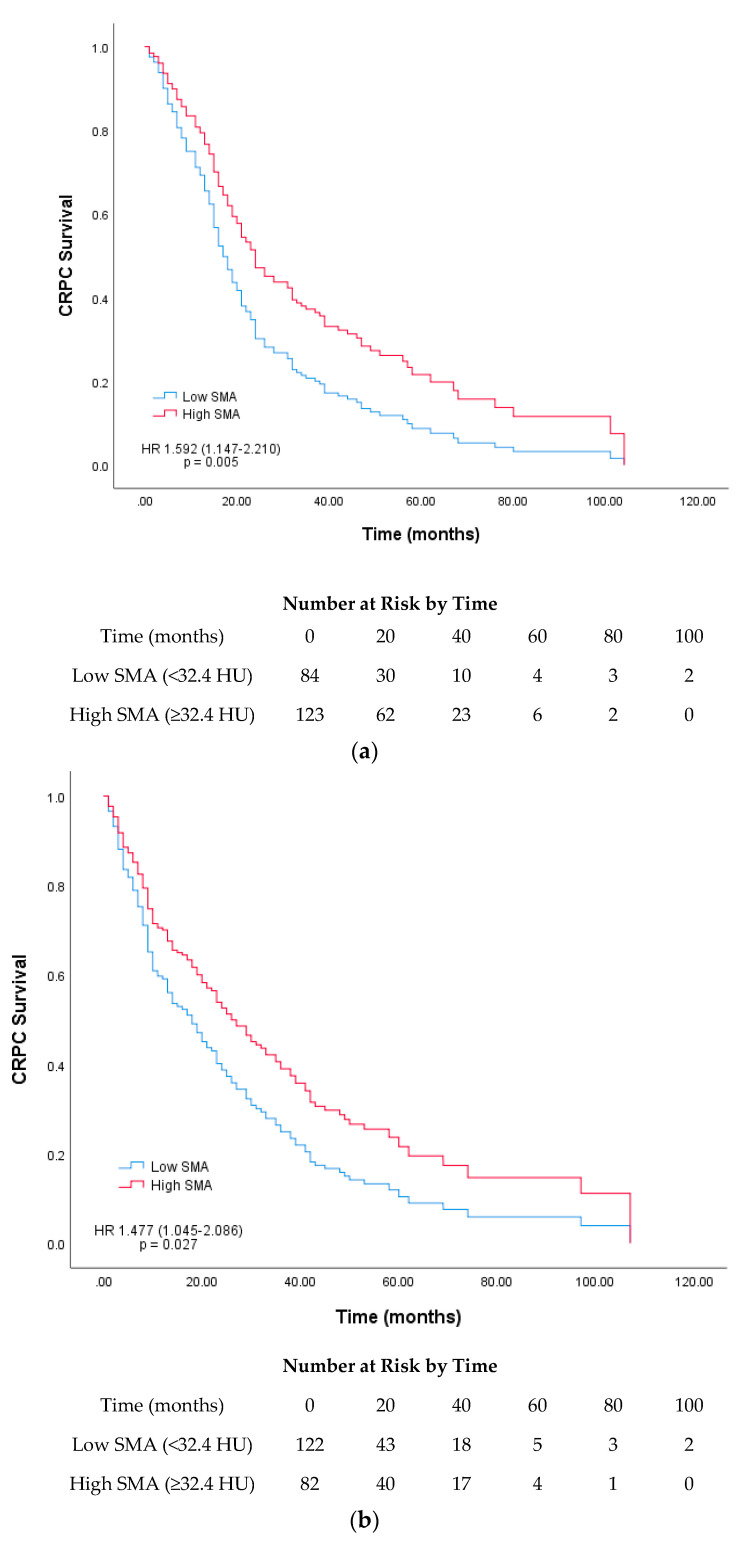
Survival curves based on Cox regression analysis of the survival of patients with castration-resistant prostate cancer (CRPC) stratified according to their median skeletal muscle attenuation (SMA; Hounsfield unit) for the age groups (**a**) <70 and (**b**) >70 years at CRPC diagnosis.

**Figure 5:**
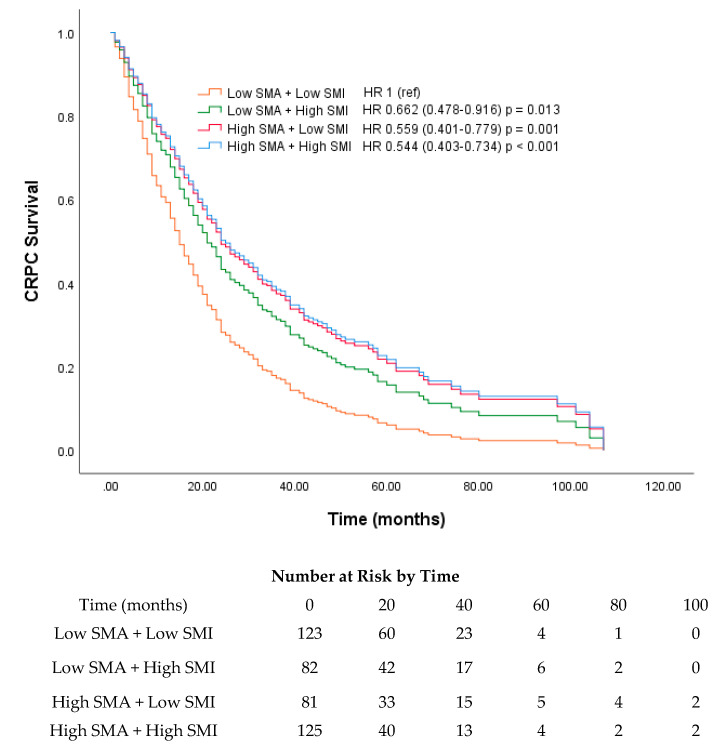
Survival curves based on Cox regression analysis of survival of patients with castration-resistant prostate cancer (CRPC) stratified according to their skeletal muscle phenotypes.

Our subgroup analysis was performed based on the median age at CRPC diagnosis (70 years). A survival curve was plotted based on the low and high SMA and SMI groups for each age group. For SMI, a significant difference was found between the low and high groups (17 vs. 29 months) in the >70-year-old age group (*p* = 0.002) (Figure 3). For SMA, both the <70- and >70-year-old age groups showed significant differences in survival [16 (low) vs. 24 months (high) and 14 (low) vs. 30 months (high); *p* = 0.005 and 0.027, respectively; Figure 4]. Among the four skeletal muscle phenotypes, the low SMA + low SMI group had the worst outcome, with statistical significance, compared with the other three groups. Between low SMA + low SMI and low SMA + high SMI groups, the latter group showed significantly longer overall survival. (*p* = 0.013, Figure 5).

## 3. Discussion

In our large cohort of patients with CRPC, representative of those with chronic cancer who underwent multimodal treatment, we found that muscle characteristics (represented by SMA and SMI) can be significant prognosticators of survival; these prognosticators were more meaningful to predict the survival of elderly patients with CRPC. To the best of our knowledge, this is the first study to analyze the relationship of prognosis in CRPC with muscle characteristics (SMA and SMI) and skeletal muscle phenotype, both of which could be obtained using routine CT; our findings suggest the importance of these characteristics in elderly patients. 

The prevalence of cancer is expected to increase because of the reduced mortality from other diseases, and the number of patients with chronic cancer is also expected to increase as a result of recent medical advances. Both muscle mass, involved in the metabolism of anticancer drugs, and fat mass, involved in the accumulation of anticancer drugs, vary among patients. Therefore, the relationships between body composition, prognosis, and cancer survival have been investigated by measuring the body composition using bioimpedance, dual-energy X-ray absorptiometry, and CT. The muscle mass obtained by CT (in particular, mass of the skeletal muscle or psoas muscle area at the L3 vertebral level) is one of the most frequently reported body composition parameters that represents the total-body skeletal muscle with high correlation and association with cancer survival [11,12].

Prostate cancer is a slow-growing carcinoma with a long duration of survival characterized by castration-naïve prostate cancer, biochemical recurrence, CRPC, and neuroendocrine differentiation during multimodal treatment. Several studies have evaluated the relationship between the body composition and survival of patients with prostate cancer [13,14,15,16]. However, muscle mass and prostate cancer survival did not appear to be correlated. Pak et al. reported a significant relationship between the PMI and prostate cancer prognosis in an Asian cohort [14]. Although a cut-off value had been used to define sarcopenia previously, Pak et al. reported differences in prognosis by dividing the PMI into quartiles. 

Patients with CRPC are usually elderly men who have received long-term ADT. Castration through ADT increases fat mass and decreases muscle mass and quality [17,18,19]. Smith et al. reported that more severe sarcopenia developed in older and longer-term recipients of ADT [16]. Chang et al. also reported that the HU measured before and after ADT (using CT) showed reduced muscle attenuation [20]. These reports suggest that patients with CRPC are more vulnerable to decreased muscle mass and quality than those with other terminal cancers, owing to the long-term use of ADT. 

Although several previous studies have investigated the correlation of patient survival with sarcopenia and myosteatosis, their definitions varied depending on the type of disease or race [5,21,22,23]. Moreover, the findings of body composition studies are difficult to apply to patients of different races and with different diseases [24]. Therefore, the cut-off values used in other studies cannot be applied to our analysis, and we chose to analyze the trend by applying the median value for each group. Large-scale studies should be performed with subgrouping of patients by disease, race, and age to permit the use of body composition values in clinical practice for precise disease management.

There were significant differences in the clinical characteristics of both groups when stratified according to the median SMA or SMI values. In factors with weight components (such as BMI and BSA), significant differences were only noted between the low and high SMI groups. Variables that appeared to be more associated with the survival rate, such as intermuscular fat area, CCI, PSA level at CRPC diagnosis, and period of ADT administration before CRPC diagnosis, showed significant differences only between the low and high SMA groups. The intermuscular fat area, rate of visceral metastasis, period of ADT administration for hormone-sensitive prostate cancer (HSPC), as well as PSA and ALP levels at CRPC diagnosis were found to be higher in the low SMA group than in the high SMA group. Although there is no clear mechanism to explain this phenomenon, it is possible that the patients in the low SMA group received shorter periods ADT for HSPC because of early disease progression with higher rates of visceral metastasis, with consequently higher PSA and ALP levels. Focusing on the treated cycles of docetaxel, significantly higher numbers of cycles were treated in the high SMA and SMI groups. This finding is consistent with those of a previous study that reported sarcopenia as a poor prognostic factor for docetaxel-treated patients with CRPC [25]. These results suggest that the SMI and SMA constitute a comprehensive representation of several biological factors that could predict patient survival; SMA appears to be a more valuable prognosticator for patients with CRPC. It is plausible that although the muscle mass decreases in patients with CRPC who have undergone ADT, more attention needs to be given to increased intramyocellular fat. In our cohort, intermuscular fat area was significantly higher in the low SMA group, possibly indicating that the amount of intramyocellular fat correlated with the intermuscular fat area, whereas the SMI groups did not show significant differences. Moreover, in our cohort, the high SMA group demonstrated a significantly higher proportion of clinical trial enrollment than the low SMA group, whereas the SMI groups did not show any significant differences. However, among the low SMA group, as high SMI showed to have significantly longer survival, SMI also seems to be a meaningful factor for prediction of survival (Figure 5). Furthermore, the effects of clinical trial enrollment on survival have previously been reported in patients with CRPC [26]. Because there are several studies on the prognosis of patients with cancer and intermuscular fat area, a comprehensive study should be conducted, and cut-off values should be defined according to the patients’ BMI.

Previous studies have reported conflicting results for the relationship between the SMI or PMI and the prognosis of prostate cancer among patients belonging to different ethnicities [24,25]. Various conclusions have been made depending on the type of disease or criteria used to define sarcopenia and myosteatosis [27]. For patients with prostate cancer, the use of ADT may result in increased intramuscular fat accumulation. Although muscle mass appeared to be maintained (i.e., minimal change was observed in the SMI), the muscle quality decreased (i.e., a marked change was observed in muscle attenuation), which possibly explains the increased intramuscular fat accumulation. In our cohort, SMI showed a significant difference in the results of univariate Cox regression analysis but not in those of the multivariate Cox regression analysis (Table 2). After subgrouping the cohort according to the median age at CRPC diagnosis (<70 or >70 years), we found that a high SMI was an important prognosticator for patients aged >70 years. Between the Asian and non-Asian populations, muscle mass was a survival prognosticator in relatively leaner and older patients. Furthermore, age was not a statistically significant prognosticator for survival based on the result of univariate or multivariate Cox regression analyses (hazard ratio [HR], 1.01; confidence interval [CI], 0.99–1.02; *p* = 0.53). The results of both univariate and multivariate Cox regression analyses showed that SMA was a statistically significant prognosticator for survival (low vs. high, 15 vs. 26 months, *p* <0.001; HR, 0.70; CI, 0.55–0.90; *p* = 0.005). After subgrouping according to the median age of 70 years, SMA was a significant prognosticator in both groups based on Cox regression analysis (<70 years: 16 vs. 24 months, *p* = 0.004; >70 years: 14 vs. 30 months, *p* = 0.02, respectively). These findings suggest that for patients aged >70 years, the functional age, which can be represented by the muscle mass, is a more influential prognosticator than the chronological age.

The recent development of various anticancer drugs has led to increased survival. However, with increased survival time and the prolonged use of anticancer drugs, increased muscle degeneration and intramuscular fat accumulation may occur, leading to sarcopenia and myosteatosis. As a result, the metabolism and detoxification rates of drugs decrease, resulting in the increased occurrences of side-effects and decline in general health [25]. These findings suggest that analyzing muscle characteristics at the time of CRPC diagnosis can help to predict CRPC prognosis. The results of the present study suggest that the SMA for patients of all ages and the SMI for those aged >70 years were more meaningful prognosticators than age itself. Moreover, the analysis of the skeletal muscle phenotype revealed that the low SMA + low SMI group had the worst outcome. These findings may aid physicians in the identification of patients who require definitive treatment or conservative management. Functional age can be considered to play a role in the decrease in physical function, which was previously difficult to measure objectively. The conventional method for determining the optimal, patient-specific drug dose using BSA is subject to changes; these variations are caused by variations in the measurement of body composition using various diagnostic devices. In the current era of precision medicine, the use of body composition parameters such as SMI and SMA may be advantageous to accurately identify patient status and may lead to better treatment decisions, especially when treating patients of varying ages and races with different carcinomas. With the development of computer science, time-consuming and arduous measurement of the body composition may be automated in the near future [7,8].

There are several limitations in this study. First, because of its retrospective design, a selection bias may be present and the research methodology itself may be a limitation. Second, not all patients received the same treatment because treatments were determined by the treating physician and were based on a patient’s disease characteristics. Third, because of differences in the ethnicity and diseases of the patients, the cut-off values used in this study should not be considered the gold-standard value for other studies. Fourth, the analysis was done only by the data at CRPC diagnosis, where comparing the differences of SMA and SMI before and after ADT may support our findings. Fifth, the image analysis program used in our study may not be available at every center; this may be considered as a technical limitation.

Despite these limitations, this study provides meaningful results because a large cohort of >400 patients with CRPC was included. Furthermore, this was the first study to identify SMA as a prognosticator for the survival of patients with CRPC and to evaluate its correlation with other clinical variables.

## 4. Materials and Methods 

### 4.1. Study Population

This was a single-center cohort study in which patients were treated by different urologists. The study was approved by the institutional review board of Yonsei University College of Medicine. (IRB 2020-1413-001) We reviewed the electronic medical records of 453 consecutive patients with prostate cancer who progressed to CRPC between March 2005 and May 2019. The patients were excluded from the analysis if they did not have an appropriate pre-contrast CT image (*n* = 23), were lost to follow-up (*n* = 9), or had an unknown cause of death (*n* = 10). 

The diagnosis of prostate cancer was established based on the results of pathological examination of trans-rectal or trans-perineal prostate biopsy specimens. Additional pathological examinations for staging were performed for the patients who underwent radical prostatectomy. The clinical cancer stage was determined according to the 8th version of the American Joint Committee on Cancer Tumor Node Metastasis system. CRPC was defined as disease progression confirmed by over two consecutive continuous increases in the serum PSA level or the appearance of new metastases detected on imaging, despite the administration of ADT and castrate levels of testosterone (≤50 ng/mL).

Treatments for patients with CRPC were determined at the physician’s discretion after full discussion with the patient. The treatment agents used included abiraterone, enzalutamide, cabazitaxel, and docetaxel. Each regimen was continued until the patient experienced intolerable side-effects or disease progression or until they refused further treatment. Serum PSA measurements were performed every 1–3 months, and CT, magnetic resonance imaging, or bone scans were performed every 2–6 months at the physician’s discretion. 

### 4.2. Anthropometric and Muscle Characteristic Assessment 

Height (m) and weight (kg) were measured when determining the amount of contrast agent to be administered for imaging. The BMI was categorized using the World Health Organization Classifications for Asian populations [28]. The BSA was calculated using the Mosteller formula [29]. 

The muscle characteristic parameters were obtained using CT findings at the time of CRPC diagnosis. With the pre-contrast CT image, the muscle and intermuscular fat areas on a single cross-sectional image at the level of the L3 vertebra were analyzed using a technique that was previously shown to have high correlation with the total-body skeletal muscle (R^2^ = 0.86) [12]. The areas with different body compositions were automatically outlined using specific HU ranges, from −29 to +150 for the muscle and from −200 to −50 for the intermuscular fat area, according to previous studies, and the outline was manually adjusted, if required [30]. An image analysis program was used for measurement of the CT images (Aquarius Intuition Viewer version 4.4.12; TeraRecon, CA, USA). The muscle characteristics were categorized as SMI (cm^2^/m^2^) and SMA (HU).

### 4.3. Cut-Off Values for Dichotomization

Considering the lack of international or regional consensus on sarcopenia or myosteatosis (according to the CT values), we divided the patients according to their median SMI and SMA values into low and high SMI and SMA groups. The skeletal muscle phenotype was determined based on the following combinations of the SMI and SMA values: high SMA + high SMI, high SMA + low SMI, low SMA + high SMI, and low SMA + low SMI [31].

### 4.4. Study Endpoints

The primary endpoint of our study was the correlation between survival and muscle characteristics (SMI and SMA) obtained using CT images. The secondary endpoint was the correlation between muscle characteristics and survival within the groups stratified by age and by skeletal muscle phenotype.

### 4.5. Statistical Analysis

Mann–Whitney *U*-tests were performed for descriptive variables [32]. The Fisher’s exact test was performed to compare the categorical variables. Survival analyses were performed using Cox regression analyses. SMI and SMA were dichotomized by their median values and referred to as low and high SMI and SMA, respectively. Statistical analyses were performed using SPSS (SPSS for Windows, version 25.0, IBM, Chicago, IL, USA). *p*-values < 0.05 were considered statistically significant.

## 5. Conclusions

SMI and SMA values can predict the prognosis of metastatic CRPC. In the era of advanced imaging techniques and computer science, the SMI and SMA may soon replace the BMI and BSA. The former may become determinants for treatment choices in the future. As unfavorable skeletal muscle phenotypes can lead to worse patient outcomes, detailed investigations are required in this area. Large-scale studies involving patients of different ethnicities, diseases, and ages are needed to further understand the criteria for selecting optimal treatment.

## Figures and Tables

**Table 1:** Clinicopathological characteristics of patients with castration-resistant prostate cancer.

Variables	Total (*n* = 411)		Low SMA (<32.4 HU) (*n* = 206)		High SMA (≥32.4 HU) (*n* = 205)		*p*-Value	Low SMI (<45.2 cm^2^/m^2^) (*n* = 207)		High SMI (≥45.2 cm^2^/m^2^) (*n* = 204)		*p*-Value
Age at CRPC diagnosis, years (IQR)	70	(65–76)	72	(67–77)	69	(63–73)	<0.001	72	(67–77)	68	(62–74)	<0.001
BMI > 23 kg/m^2^ (%)	250		117	(57)	133	(65)	0.106	88	(43)	162	(79)	<0.001
BSA, m^2^ (IQR)	1.73	(1.64–1.83)	1.72	(1.63–1.83)	1.74	(1.66–1.85)	0.137	1.7	(1.60–1.79)	1.77	(1.68–1.90)	<0.001
Intermuscular area, cm^2^ (IQR)	6.11	(4.14–8.31)	7.40	(5.30–9.51)	4.89	(3.57–6.90)	<0.001	6.21	(4.26–8.78)	6.06	(4.08–7.87)	0.328
Age inclusive CCI > 3 (%)	271		122	(59)	149	(73)	0.005	127	(61)	144	(71)	0.061
KPS > 70 (%)	370		183	(92)	187	(94)	0.441	185	(89)	185	(91)	0.701
PSA at CRPC diagnosis, ng/mL (IQR)	44.2	(12.5–159.1)	63.5	(14.3–175)	31.3	(10.8–110)	0.006	48.5	(12.4–184)	39.1	(12.3–127)	0.109
Hb at CRPC diagnosis (IQR)	12.4	(11.0–13.4)	11.9	(10.8–13.1)	12.7	(11.3–13.6)	<0.001	11.9	(10.8–13.2)	12.5	(11.3–13.4)	0.009
ALP at CRPC diagnosis (IQR)	93.0	(69.0–151.0)	108	(75–194)	86	(65–140)	0.004	103	(72–202)	89	(66–138)	0.019
Albumin at CRPC diagnosis (IQR)	4.2	(3.9–4.5)	4.1	(3.8–4.4)	4.3	(4.1–4.6)	<0.001	4.1	(3.9–4.4)	4.3	(4.0–4.6)	0.001
Initial treatment (%)							0.055					0.122
Prostatectomy	129	(31)	73	(35)	56	(27)		64	(31)	65	(32)	
Radiotherapy	13	(3)	9	(4)	4	(2)		3	(1)	10	(5)	
Hormonal therapy	269	(65)	124	(60)	145	(71)		140	(68)	129	(63)	
HSPC ADT period, months (IQR)	88	(4–168)	18	(12–39)	27	(15–43)	0.004	21	(12–39)	24	(14–45)	0.109
Docetaxel cycle (IQR)	3	(5–9)	3	(2–7)	6	(3–12)	<0.001	4	(2–7)	6	(3–11)	<0.001
Metastasis (%)												
Bone	344		173	(84)	171	(83)	0.895	179	(87)	165	(81)	0.142
Lymph node	199		104	(51)	95	(45)	0.278	90	(44)	106	(52)	0.094
Visceral	70		46	(22)	24	(12)	0.006	43	(21)	27	(13)	0.049
Overall survival (%)												
1 year	257		107	(55)	150	(81)	<0.001	116	(61)	141	(75)	0.004
2 year	127		51	(29)	76	(47)	0.001	53	(39)	74	(45)	0.005
3 year	78		33	(19)	45	(30)	0.027	33	(20)	45	(29)	0.07
Clinical trial (%)	84		33	(16)	51	(25)	0.028	43	(21)	41	(20)	0.903

Chi-square test for categorical data and Mann—Whitney U test for continuous data. Values in bold indicate statistically significant data (*p* < 0.05). SMA, skeletal muscle attenuation; SMI, skeletal muscle index; CRPC, castration-resistant prostate cancer; BMI, body mass index; BSA, body surface area; CCI, Charlson comorbidity index; KPS, Karnofsky performance score; PSA, prostate-specific antigen; Hb, hemoglobin; alkaline phosphatase; HSPC, hormone-sensitive prostate cancer; ADT, androgen deprivation therapy; IQR, interquartile range.

**Table 2:** Results of Cox regression analysis for patients with castration-resistant prostate cancer.

Variables	Univariate			Multivariate ^a^		
	HR	CI	*p*-Value	HR	CI	*p*-Value
Age at CRPC diagnosis	1.01	(0.99–1.02)	0.526			
BMI (<23 vs. ≥23 kg/m^2^)	0.60	(0.48–0.76)	**<0.001**	0.85	(0.62–1.17)	0.329
BSA for every 0.2 increase	0.71	(0.61–0.83)	**<0.001**	0.83	(0.67–1.03)	0.084
Age inclusive CCI (<4 vs. ≥4)	1.04	(0.81–1.33)	0.777			
KPS (≥70 vs. <70)	1.85	(1.24–2.78)	**0.003**	0.89	(0.54–1.46)	0.649
PSA at CRPC diagnosis for every 1.0 increase	1.00	(1.00–1.00)	**<0.001**	1.00	(1.00–1.00)	**<0.001**
Hb at CRPC diagnosis for every 1.0 increase	0.77	(0.72–0.82)	<**0.001**	0.88	(0.81–0.95)	**0.002**
ALP at CRPC diagnosis for every 1.0 increase	1.00	(1.00–1.00)	<**0.001**	1.00	(1.00–1.00)	<**0.001**
Albumin at CRPC diagnosis for every 1.0 increase	0.49	(0.38–0.63)	<**0.001**	1.02	(0.73–1.44)	0.899
Bone metastasis (no vs. yes)	1.88	(1.30–2.72)	**0.001**	1.55	(1.04–2.32)	**0.03**
Lymph node metastasis (no vs. yes)	1.43	(1.14–1.81)	**0.002**	1.56	(1.20–2.01)	**0.001**
Visceral metastasis (no vs. yes)	2.35	(1.77–3.13)	<**0.001**	2.00	(1.47–2.73)	<**0.001**
SMI (low vs. high)	0.75	(0.60–0.95)	**0.017**	1.01	(0.78–1.32)	0.92
SMA (low vs. high)	0.65	(0.52–0.83)	<**0.001**	0.70	(0.55–0.90)	**0.005**

^a^ Significant variables from the univariate analysis were included in the multivariate model. Values in bold indicate statistically significant data (*p* < 0.05); HR, hazard ratio; CI, confidence interval; SMA, skeletal muscle attenuation; SMI, skeletal muscle index; CRPC, castration-resistant prostate cancer; BMI, body mass index; BSA, body surface area; CCI, Charlson comorbidity index; KPS, Karnofsky performance score; PSA, prostate-specific antigen; Hb, hemoglobin; ALP, alkaline phosphatase; IQR, interquartile range.

## References

[1] Rawla P. (2019). Epidemiology of Prostate Cancer. World J. Oncol..

[2] Miller K.D., Siegel R.L., Lin C.C., Mariotto A.B., Kramer J.L., Rowland J.H., Stein K.D., Alteri R., Jemal A. (2016). Cancer treatment and survivorship statistics, 2016. CA Cancer J. Clin..

[3] Fearon K., Strasser F., Anker S.D., Bosaeus I., Bruera E., Fainsinger R.L., Jatoi A., Loprinzi C., MacDonald N., Mantovani G. (2011). Definition and classification of cancer cachexia: An international consensus. Lancet.

[4] Martin L., Birdsell L., Macdonald N., Reiman T., Clandinin M.T., McCargar L.J., Murphy R., Ghosh S., Sawyer M.B., Baracos V.E. (2013). Cancer cachexia in the age of obesity: Skeletal muscle depletion is a powerful prognostic factor, independent of body mass index. J. Clin. Oncol..

[5] Shachar S.S., Williams G.R., Muss H.B., Nishijima T.F. (2016). Prognostic value of sarcopenia in adults with solid tumours: A meta-analysis and systematic review. Eur. J. Cancer.

[6] Aubrey J., Esfandiari N., Baracos V.E., Buteau F.A., Frenette J., Putman C.T., Mazurak V.C. (2014). Measurement of skeletal muscle radiation attenuation and basis of its biological variation. Acta Physiol..

[7] Kim Y.J., Park J.W., Kim J.W., Park C.-S., Gonzalez J.P.S., Lee S.H., Kim K.G., Oh J.H. (2016). Computerized automated quantification of subcutaneous and visceral adipose tissue from computed tomography scans: Development and validation study. JMIR Med. Inform..

[8] Bridge C.P., Rosenthal M., Wright B., Kotecha G., Fintelmann F., Troschel F., Miskin N., Desai K., Wrobel W., Babic A. (2018). Fully-automated analysis of body composition from CT in cancer patients using convolutional neural networks. OR 2.0 Context-Aware Operating Theaters, Computer Assisted Robotic Endoscopy, Clinical Image-Based Procedures, and Skin Image Analysis.

[9] Nguyen P.L., Alibhai S.M., Basaria S., D’Amico A.V., Kantoff P.W., Keating N.L., Penson D.F., Rosario D.J., Tombal B., Smith M.R. (2015). Adverse effects of androgen deprivation therapy and strategies to mitigate them. Eur. Urol..

[10] Owen P.J., Daly R.M., Livingston P.M., Fraser S.F. (2017). Lifestyle guidelines for managing adverse effects on bone health and body composition in men treated with androgen deprivation therapy for prostate cancer: An update. Prostate Cancer Prostatic Dis..

[11] Mitsiopoulos N., Baumgartner R.N., Heymsfield S.B., Lyons W., Gallagher D., Ross R. (1998). Cadaver validation of skeletal muscle measurement by magnetic resonance imaging and computerized tomography. J. Appl. Physiol..

[12] Shen W., Punyanitya M., Wang Z., Gallagher D., St.-Onge M.-P., Albu J., Heymsfield S.B., Heshka S. (2004). Total body skeletal muscle and adipose tissue volumes: Estimation from a single abdominal cross-sectional image. J. Appl. Physiol..

[13] Fowke J.H., Motley S.S., Concepcion R.S., Penson D.F., Barocas D.A. (2012). Obesity, body composition, and prostate cancer. BMC Cancer.

[14] Pak S., Park S.Y., Shin T.J., You D., Jeong I.G., Hong J.H., Kim C.S., Ahn H. (2019). Association of Muscle Mass with Survival after Radical Prostatectomy in Patients with Prostate Cancer. J. Urol..

[15] Lee J.S., Lee H.S., Ha J.S., Han K.S., Rha K.H., Hong S.J., Chung B.H., Koo K.C. (2018). Subcutaneous Fat Distribution is a Prognostic Biomarker for Men with Castration Resistant Prostate Cancer. J. Urol..

[16] Antoun S., Bayar A., Ileana E., Laplanche A., Fizazi K., di Palma M., Escudier B., Albiges L., Massard C., Loriot Y. (2015). High subcutaneous adipose tissue predicts the prognosis in metastatic castration-resistant prostate cancer patients in post chemotherapy setting. Eur. J. Cancer.

[17] van Londen G.J., Levy M.E., Perera S., Nelson J.B., Greenspan S.L. (2008). Body composition changes during androgen deprivation therapy for prostate cancer: A 2-year prospective study. Crit. Rev. Oncol./Hematol..

[18] Smith M.R., Saad F., Egerdie B., Sieber P.R., Tammela T.L., Ke C., Leder B.Z., Goessl C. (2012). Sarcopenia during androgen-deprivation therapy for prostate cancer. J. Clin. Oncol..

[19] Haseen F., Murray L.J., Cardwell C.R., O’Sullivan J.M., Cantwell M.M. (2010). The effect of androgen deprivation therapy on body composition in men with prostate cancer: Systematic review and meta-analysis. J. Cancer Surviv..

[20] Chang D., Joseph D.J., Ebert M.A., Galvao D.A., Taaffe D.R., Denham J.W., Newton R.U., Spry N.A. (2014). Effect of androgen deprivation therapy on muscle attenuation in men with prostate cancer. J. Med. Imaging Radiat. Oncol..

[21] van Dijk D.P.J., Bakens M.J.A.M., Coolsen M.M.E., Rensen S.S., van Dam R.M., Bours M.J.L., Weijenberg M.P., Dejong C.H.C., Olde Damink S.W.M. (2017). Low skeletal muscle radiation attenuation and visceral adiposity are associated with overall survival and surgical site infections in patients with pancreatic cancer. J. Cachexia Sarcopeni. Muscle.

[22] Antoun S., Lanoy E., Iacovelli R., Albiges-Sauvin L., Loriot Y., Merad-Taoufik M., Fizazi K., di Palma M., Baracos V.E., Escudier B. (2013). Skeletal muscle density predicts prognosis in patients with metastatic renal cell carcinoma treated with targeted therapies. Cancer.

[23] Kazemi-Bajestani S.M., Mazurak V.C., Baracos V. (2016). Computed tomography-defined muscle and fat wasting are associated with cancer clinical outcomes. Semin. Cell Dev. Biol..

[24] Mason R.J., Boorjian S.A., Bhindi B., Rangel L., Frank I., Karnes R.J., Tollefson M.K. (2018). The Association Between Sarcopenia and Oncologic Outcomes After Radical Prostatectomy. Clin. Genitourin. Cancer.

[25] Ohtaka A., Aoki H., Nagata M., Kanayama M., Shimizu F., Ide H., Tsujimura A., Horie S. (2019). Sarcopenia is a poor prognostic factor of castration-resistant prostate cancer treated with docetaxel therapy. Prostate Int..

[26] Koo K.C., Lee J.S., Kim J.W., Han K.S., Lee K.S., Kim D.K., Ha Y.S., Rha K.H., Hong S.J., Chung B.H. (2018). Impact of clinical trial participation on survival in patients with castration-resistant prostate cancer: A multi-center analysis. BMC Cancer.

[27] Baracos V.E., Arribas L. (2018). Sarcopenic obesity: Hidden muscle wasting and its impact for survival and complications of cancer therapy. Ann. Oncol..

[28] (2004). Appropriate body-mass index for Asian populations and its implications for policy and intervention strategies. Lancet.

[29] Mosteller R.D. (1987). Simplified calculation of body-surface area. N. Engl. J. Med..

[30] Reinders I., Murphy R.A., Brouwer I.A., Visser M., Launer L., Siggeirsdottir K., Eiriksdottir G., Gudnason V., Jonsson P.V., Lang T.F. (2015). Muscle Quality and Myosteatosis: Novel Associations with Mortality Risk: The Age, Gene/Environment Susceptibility (AGES)-Reykjavik Study. Am. J. Epidemiol..

[31] Rodrigues C.S., Chaves G.V. (2018). Skeletal muscle quality beyond average muscle attenuation: A proposal of skeletal muscle phenotypes to predict short-term survival in patients with endometrial cancer. J. Natl. Compr. Cancer Netw..

[32] Assel M., Sjoberg D., Elders A., Wang X., Huo D., Botchway A., Delfino K., Fan Y., Zhao Z., Koyama T. (2019). Guidelines for reporting of statistics for clinical research in urology. BJU Int..

